# Real-World Effectiveness and Safety of Tildrakizumab in a Large Spanish Multicenter Cohort from Spanish Psoriasis Group (GPS)

**DOI:** 10.3390/pharmacy14030063

**Published:** 2026-04-24

**Authors:** Mar Llamas-Velasco, Mercedes Hospital, Anna López-Ferrer, Pedro Herranz, Ricardo Ruíz-Villaverde, Almudena Mateu, Francisco Javier García-Latasa, Raquel Rivera, Lourdes Rodriguez Fernández-Freire, Elena Del Alcazar, Sergio Santos, Salvador Arias, Alvaro Gónzalez-Cantero, Isabel Belinchon, Gregorio Carretero, Marta Ferran, Diana Ruiz-Genao, Noemí Eiris, Antonio Sahuquillo, Javier Mataix, Jose-María Carrascosa, Pablo de la Cueva, Laura Salgado-Boquete

**Affiliations:** 1 Department of Dermatology, Hospital Universitario de la Princesa, Instituto de Investigación Biomédica de la Princesa, Universidad Autónoma de Madrid, 28006 Madrid, Spain; 2 Department of Dermatology, Hospital Universitario Puerta de Hierro, 28222 Majadahonda, Spain; 3 Department of Dermatology, Hospital de la Santa Creu i Sant Pau, 08041 Barcelona, Spain; 4 Department of Dermatology, Hospital Universitario La Paz, 28046 Madrid, Spain; 5 Department of Dermatology, Hospital Universitario San Cecilio, Instituto Biosanitario de Granada (IBS.GRANADA), 18016 Granada, Spain; 6 Department of Dermatology, Hospital Universitario Central de Asturias, 33011 Oviedo, Spain; 7 Department of Dermatology, Hospital Universitario Royo Villanova, 50015 Zaragoza, Spain; 8 Department of Dermatology, Hospital Universitario 12 de Octubre, 28041 Madrid, Spain; 9 Department of Dermatology, Hospital Virgen del Rocío, 41013 Sevilla, Spain; 10 Department of Dermatology, Hospital Universitari Germans Trias i Pujol, 08916 Badalona, Spain; 11 Department of Dermatology, Hospital Virgen de los Lirios, 03804 Alcoy, Spain; 12 Department of Dermatology, Hospital Virgen de las Nieves Hospital, 18014 Granada, Spain; 13 Department of Dermatology, Hospital Universitario Ramón y Cajal, Instituto Ramón y Cajal de Investigación Sanitarian (IRYCIS), 28034 Madrid, Spain; 14 Faculty of Medicine, Universidad Francisco de Vitoria Madrid, 28223 Madrid, Spain; 15 Department of Dermatology, Hospital General Universitario Dr. Balmis, Departamento de Medicina Clínica, Instituto de Investigación Sanitaria y Biomédica de Alicante (ISABIAL), Universidad Miguel Hernández, 03010 Alicante, Spain; 16 Department of Dermatology, Hospital Universitario de Gran Canaria Dr. Negrín, 35010 Las Palmas de Gran Canaria, Spain; 17 Department of Dermatology, Hospital del Mar, 08003 Barcelona, Spain; 18 Department of Dermatology, Hospital Universitario Fundación Alcorcon, 28922 Madrid, Spain; 19 Department of Dermatology, Hospital Universitario Virgen Macarena, 41009 Sevilla, Spain; 20 Department of Dermatology, Hospital Universitari Doctor Peset, 46026 Valencia, Spain; 21 Hospital Marina Baixa, 03570 Alicante, Spain; 22 Department of Dermatology, Hospital Infanta Leonor, 28031 Madrid, Spain; 23 Department of Dermatology, Complejo Hospitalario Universitario de Pontevedra, 36071 Pontevedra, Spain; laurasalgado.derma@gmail.com

**Keywords:** tildrakizumab, psoriasis, real-world evidence, elderly, biological therapy

## Abstract

Background: Tildrakizumab, an anti-IL-23p19 monoclonal antibody, has demonstrated efficacy in clinical trials, but real-world evidence remains crucial for confirming its profile in diverse populations. Methods: We have conducted a multicenter, retrospective observational study within the Spanish Psoriasis Group (GPS). This study updates previous findings with a larger sample size (*n* = 372) and longer follow-up. We assessed absolute Psoriasis Area and Severity Index (PASI), Body Surface Area (BSA), and the Dermatology Life Quality Index (DLQI) improvements, as well as safety, in patients with moderate-to-severe plaque psoriasis. Results: The cohort included a large population of patients with a high prevalence of comorbidities and prior biologic exposure. Effectiveness was high, with a significant proportion of patients achieving PASI < 1. Compared to recent real-world data, our cohort demonstrates superior complete clearance rates (PASI < 1) and includes a comprehensive DLQI assessment. Notably, 79 patients were aged ≥65 years, confirming the drug’s utility in the elderly. Safety was consistent with previous reports, with no new signals detected. Conclusions: Tildrakizumab shows robust effectiveness and safety in a complex, bio-experienced real-world population. The lack of clinical predictors of response suggests a need for future pharmacogenetic exploration.

## 1. Introduction

Psoriasis is a chronic, systemic inflammatory disease, affecting 2–4% of the Western population, which significantly impacts patients’ quality of life [1].

The advent of targeted biological therapies, particularly interleukin-23 (IL-23) inhibitors, such as tildrakizumab, a high-affinity humanized monoclonal antibody targeting p19, has transformed the management of moderate-to-severe plaque psoriasis [2,3]. The therapeutic landscape of moderate-to-severe plaque psoriasis has been fundamentally revolutionized by the advent of therapies targeting the interleukin-23 (IL-23)/Th17 axis [4,5]. As highlighted in the comprehensive review by Megna et al., the specific inhibition of the p19 subunit by agents such as tildrakizumab has allowed for the effective suppression of disease-driving inflammation whilst preserving IL-12-mediated host defense mechanisms [6]. Tildrakizumab, has shown long-term efficacy and safety in the reSURFACE 1 and 2 pivotal trials improving high-burden skin symptoms, sleep quality and work productivity driven by a quick improvement in health-related quality of life (HRQol) [7,8].

This targeted approach translates into profound and sustained skin clearance, coupled with an exceptionally favorable safety profile. However, Megna and colleagues rightly emphasize that while pivotal randomized controlled trials have established the foundational efficacy of IL-23 inhibitors, these tightly controlled environments only partially reflect the heterogeneous and medically complex populations encountered in daily clinical practice [6]. Real-world patients frequently present with a multitude of comorbidities, extensive prior biologic exposure, and variable adherence profiles that can significantly influence therapeutic outcomes. However, patients in clinical trials often differ from those encountered in daily practice; indeed, up to 25% of patients may not meet standard inclusion criteria, which are often designed to exclude individuals with a higher risk of adverse events [9].

Consequently, real-world evidence (RWE) is essential to validate clinical trial findings, especially in complex populations such as those with comorbidities, active malignancies, a history of biologic failure (bio-experienced), or advanced age.

Previous studies by the Spanish Psoriasis Group (GPS) provided early insights into tildrakizumab’s performance [10]. Since then, the literature has been enriched by other regional studies [11] and data from diverse ethnic backgrounds, including Asian populations [12].

This study presents a robust updated analysis of the RWE tildrakizumab GPS cohort. We aim to describe the effectiveness and safety of tildrakizumab with an expanded sample size and extended follow-up. Furthermore, we contextualize our findings by comparing them with the recent cohort specifically highlighting outcomes in bio-experienced patients and the elderly, while addressing the challenge of identifying clinical prognostic factors. Thus, our cohort, comprising a substantial proportion of bio-experienced individuals and patients with metabolic syndrome, directly addresses the existing gap in the literature. By evaluating tildrakizumab in an unselected, real-world setting, our data echo the findings discussed by Megna et al., reaffirming that the long-term durability and safety observed in trials can be successfully mirrored in daily practice [6]. Furthermore, the capacity of IL-23 inhibitors to maintain drug survival over extended periods is particularly advantageous for patients who have previously failed alternative biologic classes, such as anti-TNF or anti-IL-17 agents. Consequently, expanding real-world evidence is paramount not only for validating clinical trial results but also for informing clinical decision-making when managing challenging patient demographics that require sustained, safe, and effective intervention.

## 2. Materials and Methods

### 2.1. Data Collection

We designed a multicenter, retrospective, observational study including moderate-to-severe patients with psoriasis from 22 hospitals treated with tildrakizumab in clinical practice. The research was conducted in accordance with the Declaration of Helsinki on Ethical Principles for Medical Research Involving Human Subjects and was approved by the Galicia-Sur local clinical research ethics committee. Patients were eligible for inclusion if they had been treated with tildrakizumab for psoriasis in routine clinical practice, were over 18 years old, and had received at least one prior dose of the biological drug. We included patients that started the drug from 1 September 2019 to 1 November 2023. The same protocol has been used in other collaborative compilations of data [10].

Anonymized data were extracted from electronic medical records. Variables included demographic characteristics (age, sex, BMI), disease duration, comorbidities (including psoriatic arthritis, cardiovascular risk factors, and history of tumors), and prior treatments. Furthermore, patterns of dose optimization in clinical practice—such as omitting the induction phase, extending administration intervals, or dose escalation—were systematically recorded.

### 2.2. Efficacy and Safety Assessment

Effectiveness was evaluated using the Psoriasis Area and Severity Index (PASI), Body Surface Area (BSA), and the Dermatology Life Quality Index (DLQI) at baseline and during follow-up visits. The primary effectiveness endpoints included the proportion of patients achieving absolute PASI < 2 and PASI < 1 at different time points: weeks 12–16, 24, 52, and 104. Safety was assessed by recording adverse events (AEs) and discontinuations. We have evaluated drug survival for up to 50 months of follow-up and analyzed variables that might influence treatment response or persistence. Reasons for drug withdrawal were classified as primary or secondary failure, safety concerns (serious AE) and others (lack of adherence, patient decision).

### 2.3. Statistical Analysis

Qualitative variables were expressed as absolute (n) and relative (percentage) frequencies. Continuous variables were expressed as mean and standard deviation (SD). Pearson’s chi-squared test was used to compare qualitative variables, and Student’s *t*-test was used for quantitative variables after verifying a normal distribution. Survival studies were performed using Kaplan–Meier analysis, and predictive factors were evaluated by Cox regression. All registered clinical variables were studied using simple and multiple logistic regression models, expressing the results as odds ratios (OR) together with their 95% confidence intervals. Variables for the multivariate models were selected based on clinical relevance and their statistical significance in the initial univariate analysis, with prior checks for multicollinearity to ensure model robustness.

**Figure 1:**
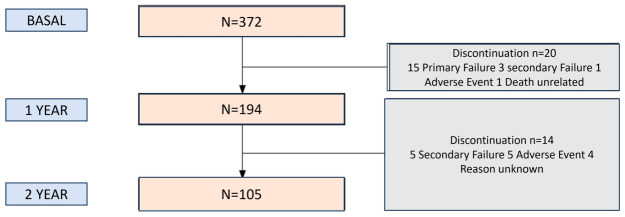
Layout of patients over time.

Given the real-world setting of this cohort, efficacy data were analyzed using the “as observed” method. This approach utilizes all available data at each specific time point without applying statistical imputation techniques. As illustrated in the patient layout over time (Figure 1), the overall number of treatment discontinuations was notably low; therefore, this approach minimizes artificial bias. Consequently, a non-responder was strictly defined as any patient who discontinued treatment for any reason, including lack of efficacy, adverse events, or patient-related decisions. This conservative definition was deliberately chosen to mimic an intention-to-treat (ITT) analysis in a real-world scenario, ensuring that the drug’s overall effectiveness is not overestimated despite the low dropout rate. The statistical analysis was performed using the SPSS software package (version 23.0 for Windows), with statistical significance set at *p* < 0.05.

## 3. Results

Baseline characteristics: The updated cohort comprised 372 patients, 54% of whom were male, with a mean age of 51.7 years (SD 15.6) and a mean weight of 82.2 kg (SD 20.8). The mean BMI was 26.8 kg/m^2^ (SD 6.2). Psoriatic arthritis was present in 12.9% of patients, with exclusively peripheral involvement in all cases. Most patients presented with early-onset psoriasis and long-standing disease, with a mean duration of 19.3 years (SD 12.0) prior to tildrakizumab initiation.

The population was characterized by a high burden of comorbidities. A total of 19.5% of patients met the criteria for metabolic syndrome, and a similar proportion (18.5%) had psychiatric comorbidities, including depression, anxiety, and schizophrenia (*n* = 3). Regarding infectious history, 21.7% had a positive QuantiFERON test. Active hepatitis B was reported in 2.4% of patients, while HIV and HCV prevalence were both 2.2%. Concomitant inflammatory bowel disease was rare (0.8%), and 6.2% of patients had a history of malignancy.

Notably, a substantial proportion of the cohort (71.8%) was bio-experienced, having failed at least one prior biological therapy; specifically, 13% had failed more than two, and 17% more than three biological agents. Regarding specific prior classes, 41.7% had received anti-TNF agents, 14.5% had used other anti-IL-23 agents (including ustekinumab), and 10.8% had exposure to anti-IL-17 inhibitors.

The treatments immediately prior to tildrakizumab are summarized as follows: most patients had received systemic therapies, including anti-TNF agents (41.7%), anti–IL-23 (14.5%), and anti–IL-17 agents (10.8%). Conventional or novel systemic treatments accounted for 28% of patients, while only 2.7% had been treated exclusively with topical therapies. When analyzing treatment sequences, patients switching from anti-TNF agents had a mean of 1.3 prior biologic lines, whereas those switching from anti-IL-23 (p19 or p40) had a mean of 1.73. Notably, patients switching from anti-IL-17 agents had a significantly higher number of prior treatment lines (mean 1.95) compared to other subgroups (*p* < 0.05). The mean number of systemic treatments prior to tildrakizumab for the entire cohort was 1.6.

**Figure 2:**
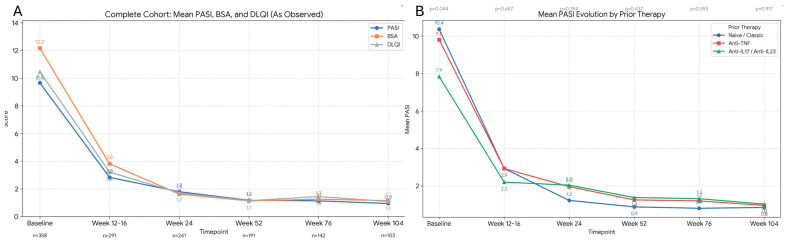
As observed analysis of effectiveness in (**A**) global cohort and (**B**) groups of patients stratified by previous treatment before tildrakizumab. *p* = 0.44 (ANOVA test) when compared means of baseline PASI in the different groups of patients stratified by previous treatment before tildrakizumab shows that cohort treated with antiIL shows a lower baseline PASI than naïve ones. All the other analysis shows no statistically significant differences.

Effectiveness baseline: Severity was assessed as usually using PASI, BSA and DLQI being the mean values for these parameters 9.6 (SD 6.1), 12.2 (SD 10.2) and 10.4 (SD 6.4). The effectiveness analysis performed in week 24 showed a decrease in mean PASI to 1.7 (SD 4.8), BSA to 1.6 (SD 4.4) and DLQI to 1.2 (SD 3.3). At week 52 85.1% and 75.8% of patient achieved PASI ≤ 2 and ≤1, with even better results in the fraction of patients reaching 104 weeks. We have also analyzed the effectiveness stratifying by the previous biologic therapy before starting and by age (>65 years vs. <65 years) (Figure 2).

**Figure 3:**
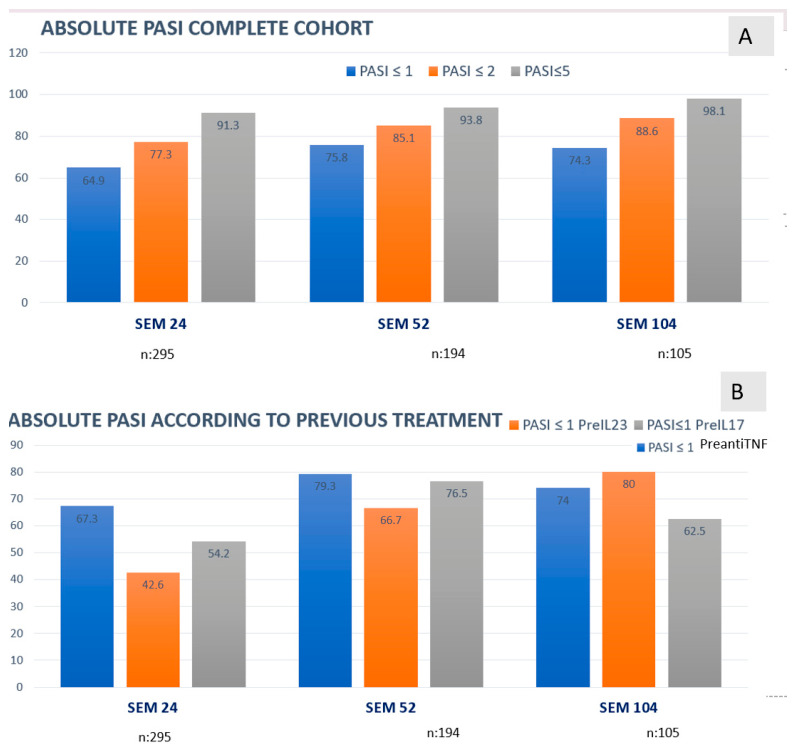
Absolute PASI in the different observation points. As observed analysis. (**A**) Complete cohort. (**B**) Stratification according to previous therapy. ANOVA test comparing the different cohorts showed non-statistically significant *p* values (*p* = 0.444 week 24; *p* = 0.375 at week 52 and *p* = 0.0576 at week 104).

Analysis of absolute PASI showed that the proportion of patients achieving PASI < 1, <2, and <5 increased over the follow-up period (Figure 3A). However, when the same analysis was performed stratifying by prior treatment before tildrakizumab, slightly lower proportions were observed in patients previously treated with anti–IL-17 and anti–IL-23 therapies compared with those who initiated tildrakizumab after anti-TNF treatment (Figure 3B).

**Figure 4:**
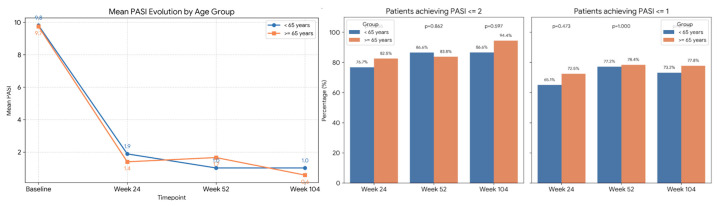
As observed mean PASI of the older than 65 and the ones younger than 65 y. Percentages of patients achieving PASI < 2 o PASI < 1 of the elderly patients compared with the rest of the cohort. *p* value 0.656 and 0.915 respectively at week 24 in PASI < 2 and week 104 in PASI < 1.

No differences in efficacy were observed among the 79 patients aged over 65 years included in the cohort (Figure 4).

The multivariate analysis revealed no differences when effectiveness was correlated with gender, obesity, psoriatic arthritis, or prior exposure to biologic therapy.

**Figure 5:**
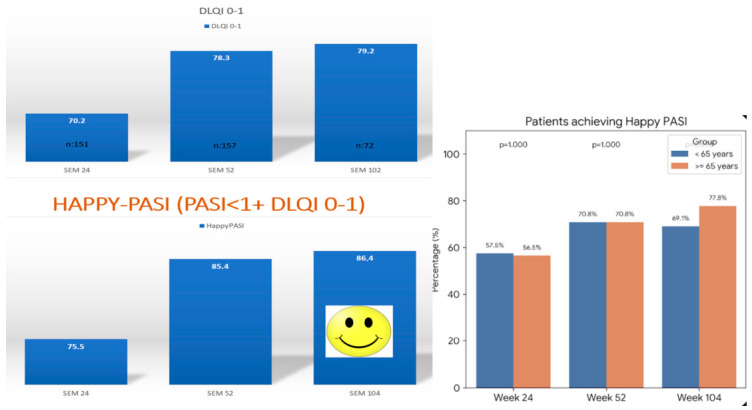
Happy-PASI is an investigator defined variable encompassing both having PASI < 1 and DLQI0/1. Happy PASI in the elderly patients compared with the younger than 65 years (no statistically significant comparisons).

Finally, regarding DLQI improvement, the proportion of patients achieving DLQI 0/1 increased up to week 52 and remained stable thereafter. When combining patients with DLQI 0/1 and concomitant PASI < 1, up to 86.4% of patients with available data achieved both outcomes at week 104 (Figure 5).

Safety among the 372 patients included, adverse events were uncommon and mostly mild. One patient (0.2%) experienced death unrelated to treatment. Infections were reported in six patients (1.6%), while other individual adverse events—arthritis, hemorrhagic stroke, local reactions, weight loss, headache, and anxiety—each occurred in one patient (0.2%).

Additionally, 81 patients (21.7%) had latent tuberculosis infection and received prophylactic treatment prior to initiating therapy. A history of neoplasia was present in 23 patients (6.2%).

### 3.1. Dose Optimization

In our cohort, 81.7% of the patients started SmPC-recommended treatment receiving induction doses of tildrakizumab 100 mg at Weeks 0 and 4 and then receiving the drug every 12 weeks. Up to 9.4% did not undergo induction. In 10.2% of patients, the treatment optimization was performed by expanding the administration interval. However, the time to perform this optimization was not registered in our cohort. One patient received tildrakizumab 200 mg from Week 4 due to insufficient response.

### 3.2. Persistence

**Figure 6:**
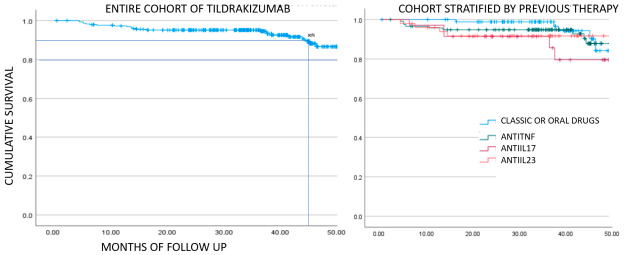
Kaplar–Meier analysis of survival of the entire cohort and stratified by previous therapy. Log-rank *p*-value 0.173.

Drug survival was excellent, with a mean duration of 50 months (95% CI: 49.350–51.957). As shown in Figure 6, 90% of patients remained on treatment for 45 months; the subsequent decrease reflects censored data, as longer follow-up information was not available. When stratified by prior therapy, there was a tendency toward slightly lower survival among patients who initiated tildrakizumab after anti–IL-17 treatment.

Baseline demographic data of our cohort can be found in Supplementary Table S1.

Previous RWE published large series on tildrakizumab can be found in Table 1.

## 4. Discussion

This multicenter study is one of the largest real-world analyses of tildrakizumab to date, confirming the drug’s robust effectiveness and safety profile initially observed in Phase III clinical trials [7,8,26,27]. Importantly, it evaluates tildrakizumab in a complex and heterogeneous patient population over a longer follow-up period, providing evidence of the drug’s high therapeutic durability even in difficult-to-treat scenarios.

Compared to the RESurface clinical trials, our cohort had more comorbidities, a higher proportion of patients with prior biologic therapy, and a lower baseline PASI (9.6 vs. 19.3–20.7 in the trials), making it essential to analyze absolute PASI scores [28]. Regarding efficacy, we observed a slightly higher response rate at week 24 compared to the clinical trials at week 28, a finding consistent with short-term series and aligned with the TILOT study (*n* = 412), which reported data up to 52 weeks [15,20,23,29]. Our data also aligns with efficacy trends seen in more recent international cohorts, such as the 28-week Chinese study [12] confirming the drug’s versatility across populations.

Notably, our data—showing 85.1% and 88.6% of patients achieving PASI ≤ 2 at weeks 52 and 104, respectively—compare favorably to the TILOT study, which reported 74.6% of patients with PASI < 3 and 88.4% with PASI < 5 at week 52 [15]. Moreover, the proportion of patients with comorbidities, including those with active neoplasms, aligns with the drug’s favorable safety profile in this challenging population, as previously described. Despite having a higher proportion of bio-experienced patients than Izu-Belloso et al., our cohort demonstrated highly favorable outcomes in terms of complete skin clearance, maintaining high rates of PASI < 1 [11].

Additionally, unlike some RWE studies where patient-reported outcomes are missing, in our cohort, the comprehensive assessment of DLQI in our study addresses a gap often found in retrospective chart reviews, demonstrating that clinical improvements translate into meaningful quality-of-life benefits not only in clinical trials [30,31,32]. This idea aligns our cohort with Gottlieb et al. recent metanalysis demonstrating the consistent, high-level benefit of tildrakizumab in routine clinical settings, [33] specifically highlighting significant improvements in quality of life parameters, as our percentage of patients achieving absolute PASI lower than 1 in highly bio-experienced individuals demonstrates robust real-world performance consistent with global observations. Our study robustly corroborates also the clinical clearance translating directing into substantial amelioration of DLQI as previously published [25,30,33]. The consistency between our national data and the international meta-analysis underscores the reproducibility of tildrakizumab’s efficacy. It confirms that the positive outcomes observed are not anomalies of a specific regional demographic, but rather inherent pharmacological benefits of the drug.

Finally, our study also adds weight to the evidence supporting tildrakizumab in geriatric population. With 79 patients aged ≥65 years, we confirmed that age and immunosenescence do not compromise safety or efficacy. This aligns with the specific findings of Liu, at 28 weeks, [12] and Mastorino et al. ag 104 weeks, [21] supporting the positioning of tildrakizumab as a highly reliable biologic option for elderly and frail patients due to its favorable safety profile and low infection risk.

An additional safety critical consideration in the long-term management of moderate-to-severe psoriasis is the overarching impact of systemic inflammation on cardiovascular health. Patients with psoriasis inherently carry an elevated burden of cardiovascular disease and metabolic syndrome, making the selection of systemic therapies a delicate balance between cutaneous efficacy and systemic safety. A recent and exhaustive systematic review and meta-analysis by Mangkorntongsakul et al. investigated the impact of biologic therapies on major adverse cardiovascular events (MACE) in patients with psoriasis [34]. Their analysis provides robust reassurance regarding the cardiovascular safety profile of targeted biologics, demonstrating that therapies such as IL-23 inhibitors do not elevate the risk of MACE and may, in fact, offer a neutral or potentially protective cardiovascular effect by comprehensively dampening systemic inflammatory cascades [35]. In the context of our multicenter cohort, this is a finding of paramount importance. A significant proportion of our patients presented with established metabolic syndrome, obesity, and other cardiovascular risk factors at baseline. The absence of newly triggered cardiovascular events or exacerbation of pre-existing metabolic conditions in our real-world population aligns seamlessly with previous literature [36,37,38]. The ability to achieve stringent psoriasis control—evidenced by high rates of absolute PASI reductions—without compromising cardiovascular safety is a crucial attribute of tildrakizumab. This reinforces the clinical rationale for prioritizing IL-23p19 inhibitors in patients with a complex cardiometabolic background, ensuring that dermatological improvements are achieved safely alongside the multidisciplinary management of the patient’s holistic health profile.

A pivotal finding of our study is the absence of robust clinical predictors of response. Despite our large sample size and multivariate analysis, we did not identify consistent baseline characteristics (such as BMI, disease duration, or number of prior therapies) that could reliably predict super-response or failure. As in previous articles, we have not found a negative impact of psoriatic arthritis history in tildrakizumab effectiveness [20,21]. This mirrors the conflicting or inconclusive search for predictors in other large series literature [19,21,22], and is contrary to Narcisi et al., that found better PASI response at week 28 in bio-experienced patients [20]. Our failure to identify definitive clinical predictors of super-response further supports the notion that while tildrakizumab is broadly effective across diverse phenotypes, the ultimate optimization of therapy may eventually require integrating pharmacogenetic profiling as have been recently tried by our group by using genetic polymorphisms (SNPs) [39].

As meticulously outlined in a recent commentary by Ruiz-Villaverde et al., while tildrakizumab offers profound efficacy for moderate-to-severe plaque psoriasis, its real-world performance is inextricably linked to intrinsic patient characteristics [40]. Factors such as obesity, high baseline disease severity, and a history of multiple prior biologic failures can theoretically blunt therapeutic responses. Ruiz-Villaverde and colleagues advocate for tailored treatment approaches, emphasizing that understanding these variables is essential for maximizing clinical outcomes. Within our Spanish multicenter cohort, the high prevalence of bio-experienced patients—many of whom had previously failed anti-TNF and anti-IL-17 agents—provided a rigorous testing ground for these individualization concepts. Despite the recalcitrant nature of our population, the sustained effectiveness of tildrakizumab observed in our study suggests that the drug possesses a therapeutic robustness capable of overcoming historically negative prognostic factors. Nevertheless, the strategic recommendations proposed by Ruiz-Villaverde et al. remain highly applicable; careful patient selection and realistic goal-setting are crucial.

This updated multicenter study represents one of the largest real-world series of tildrakizumab to date. Our findings confirm the long-term durability of response and the favorable safety profile of the drug and add new information on the drug response after previous biological therapies. We also highlight that even 100 mg dose can be very useful even in complex patients, despite 200 mg dosing even more effective in highly inflammatory phenotypes [25].

### Limitations

Our study presents several limitations inherent to its multicenter, retrospective, observational design. First, the lack of a randomized control arm precludes direct comparative efficacy conclusions or claims of superiority against other systemic therapies. Second, the reliance on real-world clinical charts introduces potential selection bias and information bias, including expected heterogeneity and missing data during the long-term follow-up. Although we managed incomplete data using an “as observed” approach and applied a conservative definition for non-responders to mitigate artificial overestimation of efficacy, these factors collectively warrant careful interpretation when generalizing our findings. Nonetheless, the substantial sample size, the long follow-up period, and the fact that the Spanish Psoriasis Group (GPS) is a highly cohesive network actively working in psoriasis research represent significant strengths that reinforce the validity and robustness of our real-world data.

## 5. Conclusions

In conclusion, this extended real-world GPs cohort affirms tildrakizumab as a highly effective and safe biologic therapy for moderate-to-severe plaque psoriasis. From a clinical perspective, these findings are particularly relevant for the management of complex patient phenotypes that are often underrepresented in clinical trials, such as the elderly, individuals with metabolic comorbidities, and highly bio-experienced patients who have failed multiple prior systemic therapies. In these challenging clinical scenarios, tildrakizumab consistently provides deep skin clearance (PASI < 1) and improvements in patient quality of life (DLQI). While this observational data firmly supports its role as a highly reliable therapeutic option in routine dermatological practice, future research integrating pharmacogenetic profiling will be essential to further optimize personalized patient selection and maximize clinical outcomes.

## Figures and Tables

**Table 1:** Compilation of previous RWE published series on tildrakizumab.

Author/Year	*n*	Dose (mg)	Follow up	Main Endpoints
Costanzo et al., 2023 [13]	177	100	24 week	PASI ≤ 3 (88.4%), PASI 75 (92.5%), PASI 90 (74.0%), DLQI 0/1 (70.4%)
Heim et al., 2023 [14]	55	100	28 week	PASI 90 (55.8%), reduction PASI (82.1%), BSA, sPGA, safety
Tsianakas et al., 2023 [15]	412	100	52 week	PASI < 3 (74.6%), PASI < 5 (88.4%), DLQI 0/1 (48.2%), PGA scalp/nails
Berenguer-Ruiz et al., 2023 [10]	190	100	24 week	PASI ≤ 3 (87.1%), PASI ≤ 1 (40.3%), reduction PASI (88.8%), DLQI, BSA, safety
Drerup et al., 2022 [16]	150	100	76 week	Improvement PASI, BSA, DLQI, itch
Caldarola et al., 2022 [17]	59	100	28 week	PASI < 3 (79.7%), PASI 75 (81.4%), PASI 90 (64.4%),
Galán-Gutiérrez et al., 2022 [18]	14	100	16 week	PASI 75 (85.7%), PASI 90 (57.1%), PASI 100 (28.6%),
Ruiz-Villaverde et al., 2022 [19]	41	100	52 week	PASI 75 (87.8%), PASI 90 (70.7%), PASI 100 (39.0%),
Narcisi et al., 2023 [20]	237	100	52 week	PASI 75 (90.9%), PASI 90 (73.6%), PASI 100 (58.7%), PASI ≤ 2 (85.9%),
Mastorino et al., 2025 [21]	53	100	28 week	PASI 75 (81.1%), PASI 90 (62.3%), PASI 100 (41.5%), Efficacy also in bio-experienced
Ruggiero et al., 2023 [22]	42	100	52 week	PASI 75 (90.5%), PASI 90 (71.4%), PASI 100 (47.6%),
Burlando et al., 2021 [23]	39	100	52 week	PASI 100 (71.2% a 36 w), Best efficacy in bionaive
Heim et al., 2024 [24]	55	100	64 week	PASI 75 (87.0%), PASI 90 (56.5%), PASI 100 (32.6%),
Cacciapuoti et al., 2025 [25]	34	100	36 week	PASI, BSA, DLQI, Skindex-16, icth
Izu-Belloso et al., 2025 [11]	212	100 mg 8 patients 200 mg	52 week (76 w subset)	68.6% of Patients PASI < 3 at week 52 85.6% retention 104 w
Our cohort	372	100 mg 1 patient 200 mg	102 w (efficacy) 200 w subset survival	PASI < 1 75.8% 1 y; 74.3% 2 y. PASI < 1 after antiIL23 66.7% 1 y; 80% 2 years. 86.4% DLQI0/1 + PASI < 1; and 90% of survival after 45 months (almost 4 years)

## Data Availability

The data presented in this study are available on request from the corresponding author due to privacy or ethical restrictions.

## Supplementary Materials

The following supporting information can be downloaded at: https://www.mdpi.com/article/10.3390/pharmacy14030063/s1, Table S1: Demographic data of the cohort.
