# Supplementary material for: Real-World Effectiveness and Safety of Tildrakizumab in a Large Spanish Multicenter Cohort from Spanish Psoriasis Group (GPS)
Source: Pharmacy (Basel). 2026 Apr 24;14(3):63. doi: 10.3390/pharmacy14030063 (PMC13214649; doi:10.3390/pharmacy14030063)
Supplement: Supplementary file 1 [file pharmacy-14-00063-s001.zip › pharmacy-4183266-supplementary.pdf]

Table S1. Demographic data of the cohort

Characteristic	*Value (n = 372)*
Demographics	
Age (years), mean ± SD	*51.7 ± 15.6*
Male gender, %	*54%*
Weight (kg), mean ± SD	*82.2 ± 20.8*
BMI (kg/m^2), mean ± SD	*26.8 ± 6.2*
Disease Characteristics	
Psoriasis duration (years), mean ± SD	*19.3 ± 12.0*
Psoriatic Arthritis (peripheral), %	*12.9%*
Comorbidities & Infectious History	
Metabolic Syndrome, %	*19.5%*
Psychiatric comorbidities, %	*18.5%*
Positive QuantiFERON test, %	*21.7%*
History of malignancy, %	*6.2%*
Active Hepatitis B, %	*2.4%*
HIV infection, %	*2.2%*
HCV infection, %	*2.2%*
Inflammatory Bowel Disease (IBD), %	*0.8%*
Prior Treatment History	
Mean systemic treatments prior to tildrakizumab	*1.6*
Bio-experienced (≥ 1 prior biologic), %	*71.8%*
Failed > 2 prior biologics, %	*13%*
Failed > 3 prior biologics, %	*17%*
Treatments Immediately Prior to Tildrakizumab	
Anti-TNF agents, %	*41.7%*
Conventional/novel systemic treatments, %	*28%*
Anti-IL-23 agents (incl. ustekinumab), %	*14.5%*
Anti-IL-17 inhibitors, %	*10.8%*
Exclusive topical therapies, %	*2.7%*
Mean Prior Biologic Lines by Preceding Therapy	
Switching from Anti-IL-17	*1.95*
Switching from Anti-IL-23	*1.73*
Switching from Anti-TNF	*1.3*
